# The Effect of Adjunct Telephone Support on Adherence and Outcomes of the Reboot Online Pain Management Program: Randomized Controlled Trial

**DOI:** 10.2196/30880

**Published:** 2022-02-03

**Authors:** Tania Gardner, Regina Schultz, Hila Haskelberg, Jill M Newby, Jane Wheatley, Michael Millard, Steven G Faux, Christine T Shiner

**Affiliations:** 1 Clinical Research Unit for Anxiety and Depression, St Vincent's Hospital Sydney Australia; 2 Department of Pain Medicine, St Vincent's Hospital Sydney Australia; 3 Royal Ryde Rehabilitation Hospital Sydney Australia; 4 University of New South Wales Sydney, NSW Australia

**Keywords:** chronic pain, online pain management, telephone support, clinician guidance, adherence, digital health, eHealth, internet interventions, multidisciplinary

## Abstract

**Background:**

Internet-based treatment programs present a solution for providing access to pain management for those unable to access clinic-based multidisciplinary pain programs. Attrition from internet interventions is a common issue. Clinician-supported guidance can be an important feature in web-based interventions; however, the optimal level of therapist guidance and expertise required to improve adherence remains unclear.

**Objective:**

The aim of this study is to evaluate whether augmenting the existing Reboot Online program with telephone support by a clinician improves program adherence and effectiveness compared with the web-based program alone.

**Methods:**

A 2-armed, CONSORT (Consolidated Standards of Reporting Trials)–compliant, registered randomized controlled trial with one-to-one group allocation was conducted. It compared a web-based multidisciplinary pain management program, Reboot Online, combined with telephone support (n=44) with Reboot Online alone (n=45) as the control group. Participants were recruited through web-based social media and the This Way Up service provider network. The primary outcome for this study was adherence to the Reboot Online program. Adherence was quantified through three metrics: completion of the program, the number of participants who enrolled into the program, and the number of participants who commenced the program. Data on adherence were collected automatically through the This Way Up platform. Secondary measures of clinical effectiveness were also collected.

**Results:**

Reboot Online combined with telephone support had a positive effect on enrollment and commencement of the program compared with Reboot Online without telephone support. Significantly more participants from the Reboot Online plus telephone support group enrolled (41/44, 93%) into the course than those from the control group (35/45, 78%; *χ*^2^_1_=4.2; *P*=.04). Furthermore, more participants from the intervention group commenced the course than those from the control group (40/44, 91% vs 27/45, 60%, respectively; *χ^2^*_1_=11.4; *P*=.001). Of the participants enrolled in the intervention group, 43% (19/44) completed the course, and of those in the control group, 31% (14/45) completed the course. When considering the subgroup of those who commenced the program, there was no significant difference between the proportions of people who completed all 8 lessons in the intervention (19/40, 48%) and control groups (14/27, 52%; *χ^2^*_1_=1.3; *P*=.24). The treatment efficacy on clinical outcome measures did not differ between the intervention and control groups.

**Conclusions:**

Telephone support improves participants’ registration, program commencement, and engagement in the early phase of the internet intervention; however, it did not seem to have an impact on overall course completion or efficacy.

**Trial Registration:**

Australian New Zealand Clinical Trials Registry ACTRN12619001076167; https://anzctr.org.au/Trial/Registration/TrialReview.aspx?ACTRN=12619001076167

## Introduction

### Background

The global prevalence of chronic pain is estimated to be 10% to 33% [1-3], and pain conditions continue to be a leading cause of worldwide disability [4]. The accepted best practice for managing chronic pain is a multidisciplinary pain program that involves attending a group program—typically convened face to face at a hospital or clinical site—facilitated by members of a multidisciplinary team [5].

A main challenge to the management of chronic pain is the lack of services available within existing health care systems. Access to multidisciplinary pain management groups can be compromised for people living in rural areas, those with family or work commitments, and those with mental health issues that limit their ability to engage and be involved in a social group environment [6]. Furthermore, because of the COVID-19 global pandemic, increased social isolation and reduced accessibility of face-to-face clinical interactions have further compromised access to required pain management services for many people [7]. Web-based treatment programs present a solution for providing access to pain management for those unable to access clinic-based multidisciplinary pain programs [6,7] and for delivering care at reduced personnel costs [7-9].

The evidence to date suggests that internet-based health interventions that provide a combination of information and some form of user support—such as decision management support, behavior change support, or social support—lead to improvements in knowledge as well as behavioral and clinical outcomes [8,10-13]. Generally, internet-based pain programs focus on unimodal interventions that offer a single discipline of therapy, most commonly psychological therapies such as internet-delivered cognitive behavioral therapy (CBT) [14]. These programs typically show small to moderate effects on pain-related disability and interference as well as pain catastrophizing and are a cost-effective alternative to face-to-face therapy [13,14].

Reboot Online was one of the first internet-based pain management programs that offered a multidisciplinary model. The program has been shown to significantly improve pain self-efficacy and reduce pain-related disability, kinesiophobia, and psychological distress compared with usual care [15,16]. These studies were carried out within a strict clinical trial paradigm. Although highly effective for those participants who completed the program, adherence rates were moderate, with only 61% of the trial participants completing all the lessons. Adherence (completion of all 8 lessons of the program) and effectiveness (significant changes in clinical outcome measures) of the program in real-world conditions were subsequently evaluated [17]. Without the regulated framework and rigor embedded within a clinical trial design, the adherence to the Reboot Online program reduced to 41% when used under routine care conditions.

Attrition, or dropout of study participants, is a common problem for internet-based treatments [18,19]. In a systematic review of internet interventions for chronic pain [19], the range in attrition levels across published trials was considerable, from 4% to 54%. Different methods have been used to prevent dropout, such as telephone support, personalized reminders and feedback, and financial incentives [19]. However, it remains unclear how helpful these methods are [19].

Clinician-supported guidance can be an important feature in web-based interventions for mental health disorders; however, the optimal level and pattern of therapist guidance required to improve adherence remains unclear [20]. Having human social support, whether it be peer-led or specialized clinician–led, has been suggested to help support healthy behavior change [21,22] and has been shown to have small effects on improving adherence compared with unguided interventions for individuals with chronic pain [22-25]. The addition of clinician-supported guidance has not yet been tested in routine care using the Reboot Online program.

### Objectives

We aim to evaluate whether augmenting the existing Reboot Online program with telephone support by a clinician improves program adherence and effectiveness compared with the web-based program alone. We hypothesized that the Reboot Online plus telephone support group would have better adherence (defined as higher number of enrollments, higher number of commencements, and higher completion rates) to the program than the control group who received the Reboot Online program only.

## Methods

### Study Design

The study was a 2-armed, CONSORT (Consolidated Standards of Reporting Trials)–compliant, registered randomized controlled trial with one-to-one group allocation. It compared a web-based multidisciplinary pain management program, *Reboot Online*, combined with telephone support with *Reboot Online* alone as the control group.

The study was approved by the human research ethics committee of St Vincent’s Hospital, Sydney, Australia (2019/ETH08682). The trial was prospectively registered on the Australian New Zealand Clinical Trials Registry (ACTRN12619001076167), and the protocol was followed as per the registry.

### Participants

Participants were recruited between September 2019 and April 2020 through social media advertisements (eg, Facebook and Twitter) and through the This Way Up service provider network. To replicate real-world conditions, the trial was run through the established web-based platform through which the Reboot Online program is available: *This Way Up* [26]. Applicants completed a 2-step screening process. All applicants first completed web-based screening questionnaires about their chronic pain symptoms and demographic details, followed by a telephone interview to confirm their chronic pain diagnosis and study eligibility. The Mini International Neuropsychiatric Interview (version 5.0.0) [27] for major depressive disorder (MDD) and risk assessment modules were also administered during the telephone interview as a screening and diagnostic tool for depression. MDD helps us to compare the sample characteristics with our previous trials and establish the number of individuals who had comorbid depression, which also assists in clinical support. MDD plus risk assessment modules help establish how the clinician will prioritize and triage calls as well as monitor and ensure that participants who are at risk are appropriately supported throughout the program.

Applicants were eligible for inclusion if they (1) had experienced pain for >3 months, (2) were aged ≥18 years, (3) were a resident of Australia, (4) had their pain assessed by a physician in the past 3 months, (5) were prepared to provide contact details of their general practitioner, (6) had access to the internet and computer or tablet and deemed themselves to have basic computer literacy, and (7) were fluent in written and spoken English. Applicants were excluded if they had psychosis, bipolar disorder, were actively suicidal (eg, demonstrated intent or a plan or had a recent suicide attempt). These exclusion criteria were included to be consistent with other trial exclusion criteria and to be able to adequately evaluate participants’ needs and assist their access to more intensive clinical support if required. Participants were also excluded if they had participated in a group-based pain management program within the last 6 months so that their prior treatment or learning would not confound the results. Participants were also ineligible for the study if they had surgical procedures scheduled in the 6 months after their application; had commenced or made significant changes to their psychotropic medication in the previous 2 months before intake assessment; or had commenced face-to-face CBT within 4 weeks of the intake assessment. These exclusion criteria were included because of their potential confounds. We wanted to explore the effects of clinician support on program adherence and outcomes and minimize the confounding effects of these features.

Participants provided electronic informed consent before being enrolled in the study; they also provided permission for their pain physician (or general practitioner) to be notified by the study team detailing their involvement in the trial. Measures of psychological distress (Kessler-10 Psychological Distress Scale [28,29]) were monitored throughout the program for any risk of harm.

### Randomization

The eligible participants were randomly assigned to either the intervention or control group in a 1:1 allocation based on a random number sequence generated [30]. An individual web-based link for baseline data questionnaires through REDCap (Research Electronic Data Capture [31]) was sent to the eligible participants to complete. Once the initial baseline questionnaires were completed, an individual registration link to Reboot Online through the usual *This Way Up* process was sent to the participants, which gave them access to register for the course, complete pretreatment questionnaires, and commence the first lesson. Telephone screening interviewers at baseline remained blinded to the participants’ group allocation. Study staff members conducting the telephone support intervention could not be blinded to group allocation. Group allocation was indicated to the participant by whether they received telephone support after registration into the program.

### Intervention

#### Reboot Online Program (Control)

Participants received free access to the standard Reboot Online program [32]. Details of the content and design of the program have been previously outlined [16]. In brief, Reboot Online consists of 8 lessons delivered over 16 weeks. Participants follow an illustrated story of a fictional character who learns to self-manage her chronic pain using a multidisciplinary approach. The content delivers psychoeducation on the socio-psycho-biomedical nature of chronic pain within a multidisciplinary framework. The core lessons are combined with a *movement station* section: a graded, patient-led exercise program focusing on activity and exercise reactivation. This is combined with pacing: an activity-management technique aimed to maximize a person’s activity by developing a structured, slow, and gradual planned increase in activity levels over a period of time. The level of activity is dependent on the planned quantity rather than pain intensity experienced by the patient. The program also incorporates SMART (specific, measurable, achievable, relevant, and timely) goal-setting principles, whereby the patient leads the process of identifying issues or problems that matter most to them, sets the goal that they want to accomplish, and develops strategies and goals that are specific, measurable, achievable, relevant, and within a time frame. This is coupled with evidence-based CBT skills activities, including thought challenging, activity planning, problem solving, effective communication, and flare-up management. Participants also have access to expert educational videos as well as tai chi and relaxation audio guides. See Table 1 for details of the lesson content of the program. All participants received automatic email communication to notify them when a lesson was available and to encourage lesson completion as well as engaging in activity.

**Table 1:** Reboot Online lesson content.

Lesson title	Lesson content	Homework activities	Lesson resources (PDF or video)
Lesson 1: What Is Chronic Pain and What Is the Best Way to Manage It?	Chronic pain explained The Brick Wall of Chronic Pain Medication overview The movement station	Review of acute vs chronic pain Complete own Brick Wall of Chronic Pain Instructions for the movement station	Welcome (video) Medication management (video)
Lesson 2: Goal-Setting and Moving Toward Acceptance	Scans, test results, and pain The cycle of chronic pain Moving toward acceptance SMART^a^ goals	Reviewing the cycle of pain Identifying acceptance, change, and goals Setting short- and long-term SMART goals	Medical imaging (video) Making life changes (PDF)
Lesson 3: Movement, Pacing, and Daily Activity Scheduling	Exploring the relationship between pain and activity Learning about the “Boom Bust” pattern Fear avoidance beliefs Importance of pacing Introduction to daily activity scheduling	Identifying healthy vs unhealthy coping skills Pacing activity Keeping a movement diary	Daily activity scheduling (PDF)
Lesson 4: Monitoring Thoughts and Recognizing Unhelpful Thinking Patterns	Revision of chronic pain cycle and the link among thoughts, feelings, and behaviors Recognizing unhelpful thinking patterns Monitoring thoughts and learning to think more helpful thoughts	Ways to recognize own unhelpful thinking patterns Complete thought record Continue to monitor and track thoughts	—^b^
Lesson 5: Mood and Pain, Thought Challenging, and Managing Arousal	Challenging unhelpful thinking Activity planning in practice Emotions and pain Strategies to manage anxiety Mood	Thought-challenging situations Activity planning and monitoring Managing anger: looking out for triggers and learning strategies Practicing relaxation	My Thought Challenging Worksheet (PDF)
Lesson 6: Stress Management and Getting Better Sleep	What is stress? How to manage stress better Using problem solving Barriers to good sleep with chronic pain and ways to get better sleep	Recognizing sources of stress and own signs and symptoms Structured problem-solving task Sleep diary to improve habits	Good sleep guide (PDF) Better sleep for chronic pain (video)
Lesson 7: Communication and Relationships	What is good communication? Communication styles Relationships with others Ways to improve those relationships	Communication skills practice Task: select a relationship and explore how you would like that relationship to be different Revisiting thought-challenging exercise	Conversation skills tips (PDF) Chronic pain and information for family and friends (PDF)
Lesson 8: Managing Flare-ups and Continuing Management of Chronic Pain	Exploring flare-ups, relapse, and the need for a flare-up plan Continuing chronic pain management How to get further help	Ten things to help during a relapse Flare-up prevention plan Summary	What is a pain clinic? (video) Congratulations (video)

^a^SMART: specific, measurable, achievable, relevant, and timely.

^b^Not available.

#### Reboot Online Program With Telephone Support (Intervention)

Participants received free access to the standard Reboot Online program, as outlined in the control intervention, in conjunction with telephone support. They received a phone call every fortnight for the duration of the program (maximum of 8 phone calls). There was a set maximum of 3 attempts if contact by phone was unsuccessful. The phone call was conducted by a single clinician (senior allied health clinician) experienced in the management of chronic pain. To replicate what may occur in routine clinical care, we sought a flexible approach whereby the duration of the phone call was led by the needs of the patient. During the call, the participants were advised that the call was to check in and to see how their program was going. They were asked to report on their progress and encouraged to continue engaging with the program. The participants were also given the opportunity to discuss any challenges or hurdles they were experiencing, to problem-solve possible strategies to overcome them, and to receive feedback on their progress.

For both treatment groups, a senior clinician (senior pain physiotherapist TG and pain psychologist JW) experienced in chronic pain management monitored questionnaire responses, and if any responses indicated deterioration in well-being, phone contact was made with the participant and further clinical intervention was advised if indicated.

### Outcome Measures

Participants were assessed using a suite of outcome measures, collected at three time points: baseline (immediately before commencing treatment), after treatment (weeks 16-17) and follow-up (3 months after completing posttreatment questionnaires).

The primary outcome for this study was adherence to the Reboot Online program. Adherence was quantified through three metrics: (1) completion of the program assessed through the number of participants who completed all 8 lessons of the intervention and who were classified as *completers*, whereas those who did not complete all 8 lessons were classified as *n*o*ncompleters*; (2) the number of participants who enrolled to undertake the program; and (3) the number of participants who commenced at least one lesson of the program. The rate of overall program completion was the primary outcome measure for adherence. These adherence measures were chosen to capture three key potential points where patients may drop out: (1) enrolling after being prescribed the course, (2) after completing baseline questionnaires and commencing the course, and (3) once engaged in the program after each lesson. Data on adherence were collected automatically through the This Way Up platform.

The secondary outcomes were as follows:

1. Pain Self-Efficacy Questionnaire [33] to assess participants’ confidence to perform activities while in pain, with higher scores indicating greater confidence in functional capacity. A minimally clinically important difference (MCID) was considered to be 5.5 [34].
2. Tampa Scale for Kinesiophobia [35] to measure fear and avoidance of movement. An MCID was considered to be 6 [36].
3. Brief Pain Inventory [37] to assess pain severity and its impact on function through its 2 subscales for severity and pain interference. An MCID was considered to be 2 [38].
4. Pain Disability Index [39] to assess the degree to which chronic pain interferes with participants’ daily activities and essential life activities. An MCID was considered to be between 8.5 and 9.5 [28].
5. International Physical Activity Questionnaire [29,40] to measure self-reported physical activity in the previous 7 days. Metabolic equivalents and level of physical activity were calculated using a preformed Microsoft Excel program [41]. This program calculated metabolic equivalents and levels of physical activity as low, moderate, and high according to International Physical Activity Questionnaire short form categorical scoring.
6. Kessler-10 Psychological Distress Scale [42,43] to measure psychological distress.

Self-reported data on clinical outcome measures were collected through the This Way Up platform as well as the REDCap tool hosted at St Vincent’s Hospital, Sydney [44,45]. REDCap is a secure, web-based software platform designed to support data capture for research studies, providing (1) an intuitive interface for validated data capture, (2) audit trails for tracking data manipulation and export procedures, (3) automated export procedures for seamless data downloads to common statistical packages, and (4) procedures for data integration and interoperability with external sources.

### Telephone Support Time and Satisfaction

Descriptive data pertaining to the amount of clinician contact and time spent conducting the telephone support calls were collected for all participants in the intervention group. Descriptive data on treatment satisfaction and perceived usefulness of the intervention were collected from all participants (both groups) during the posttreatment assessments. Additional user feedback was collected from the intervention group regarding how helpful and motivating they found the telephone support calls received as part of the intervention. Data on subjective participant feedback were quantitative in nature (collected on a rating scale from 1 to 10) and were analyzed descriptively through median values and IQRs (because of nonparametric data distribution).

### Statistical Methods

Before commencement of the study, a power calculation was conducted to determine the minimum sample size required to detect a difference in the proportion of participants adhering to the course between the intervention and control groups. This was based on previous investigations [16], where 60% of the participants were observed to have adhered to the complete program under *control* conditions and an increase of 25% in adherence was predicted for the telephone support intervention group. Assuming a power of 80% and a Cronbach *α* set at .05, a minimum sample size of 39 participants in each group was needed to detect a 25% difference in the proportion of completers. To account for an anticipated approximate 10% dropout rate, a total of 44 participants were required in each study arm.

Descriptive statistics were used to characterize the demographic and clinical features of the study groups at baseline. Data on the time taken to deliver the telephone support and subjective participant feedback on the telephone support and web-based aspects of the Reboot Online program were also analyzed descriptively.

Cross-tabulations were used to examine between-group differences in the primary outcome of adherence. Chi-square analyses were performed with binary factors of group (intervention and control) and adherence (yes or no) for each of the identified adherence metrics (enrollment, course commencement, and course completion). A Mann–Whitney *U* test was used to compare the median number of lessons completed by the intervention and control groups.

To examine treatment efficacy, intention-to-treat linear mixed models were implemented separately for each of the secondary outcome measures. This form of analysis can robustly account for the unbalanced nature of repeated measures data, including missing data because of participant dropout. Each model included group, time, and a group-by-time interaction as fixed factors. A random effect of participant was also included in each model, with an identity covariance structure used to model the covariance structure of the random intercept. Model fit indices supported the selection of an unstructured covariance matrix for each of the outcome measures. Significant treatment effects were followed with pair-wise comparisons of their estimated marginal means. For each outcome, the estimated marginal means derived from the model were used to calculate within-group effect sizes (Hedges g, adjusted for the correlation among time points). Pre- to posttreatment effect sizes (Hedges g, adjusted for the correlation among time points) were calculated, corresponding to the changes between pre- and posttreatment values and between posttreatment and follow-up values. Between-group effect sizes (Hedges g, adjusted for the correlation among time points) for each outcome after treatment and at follow-up were also calculated. Effect sizes of <0.49, 0.50-0.79, and >0.80 were considered to be small, moderate, and large, respectively [46].

Statistical analyses were conducted using SPSS software (version 25; IBM Corp), and results were considered significant where *P*<.05.

## Results

### Baseline Sample Characteristics

Baseline and sample characteristics are outlined in Table 2*.* Demographic data were available for all participants (N=89) who met the inclusion criteria. The participants had a mean age of 49.3 years (SD 16.1; range 21-86 years), and most of them were women (59/89, 66%). At baseline, 42% (37/89) of the participants reported taking simple analgesia for pain (nonopioid analgesics such as paracetamol and nonsteroidal anti-inflammatory drugs), 27% (24/89) took gabanoids, 43% (38/89) took antidepressant medication, and 44% (39/89) took opioid analgesia. Approximately 1 in 3 participants (28/89, 32%) met the criteria for MDD at the time of study enrollment.

**Table 2:** Baseline demographics (N=89).

Characteristics			Total sample (N=89)		Intervention (n=44)		Control (n=45)		Between-group comparison				
									Values			*P* value	
Age (years), mean (SD)			49.3 (16.1)		48.0 (15.8)		50.6 (16.4)		t_84_=–0.74			.46	
**Gender, n (%)**										*χ*^2^_2_=2.2			.34
	Male	28 (32)		13 (30)		15 (33)							
	Female	59 (66)		29 (66)		30 (67)							
	Nonbinary	2 (2)		2 (5)		0 (0)							
**Rural status, n (%)**										*χ*^2^_1_=0.9			.17
	Major city	52 (58)		29 (66)		23 (51)							
	Regional or remote	37 (42)		15 (34)		22 (49)							
Major depressive disorder, n (%)			28 (32)		16 (36)		12 (27)		*χ*^2^_1_=1.0			.33	
Opioid analgesia, n (%)			39 (44)		14 (33)		25 (56)		*χ*^2^_1_=4.7			*.03* ^a^	
Antidepressants, n (%)			38 (43)		20 (47)		18 (40)		*χ*^2^_1_=0.4			.54	
Anticonvulsants, n (%)			24 (27)		13 (30)		11 (24)		*χ*^2^_1_=0.4			.54	
Benzodiazepines, n (%)			16 (18)		8 (19)		8 (18)		*χ*^2^_1_=0.0			.92	
Simple analgesia, n (%)			44 (49)		18(41)		26 (58)		*χ*^2^_1_=2.5			.11	

^a^Italicized results are significant at *P*<.05.

### Adherence

**Figure 1:**
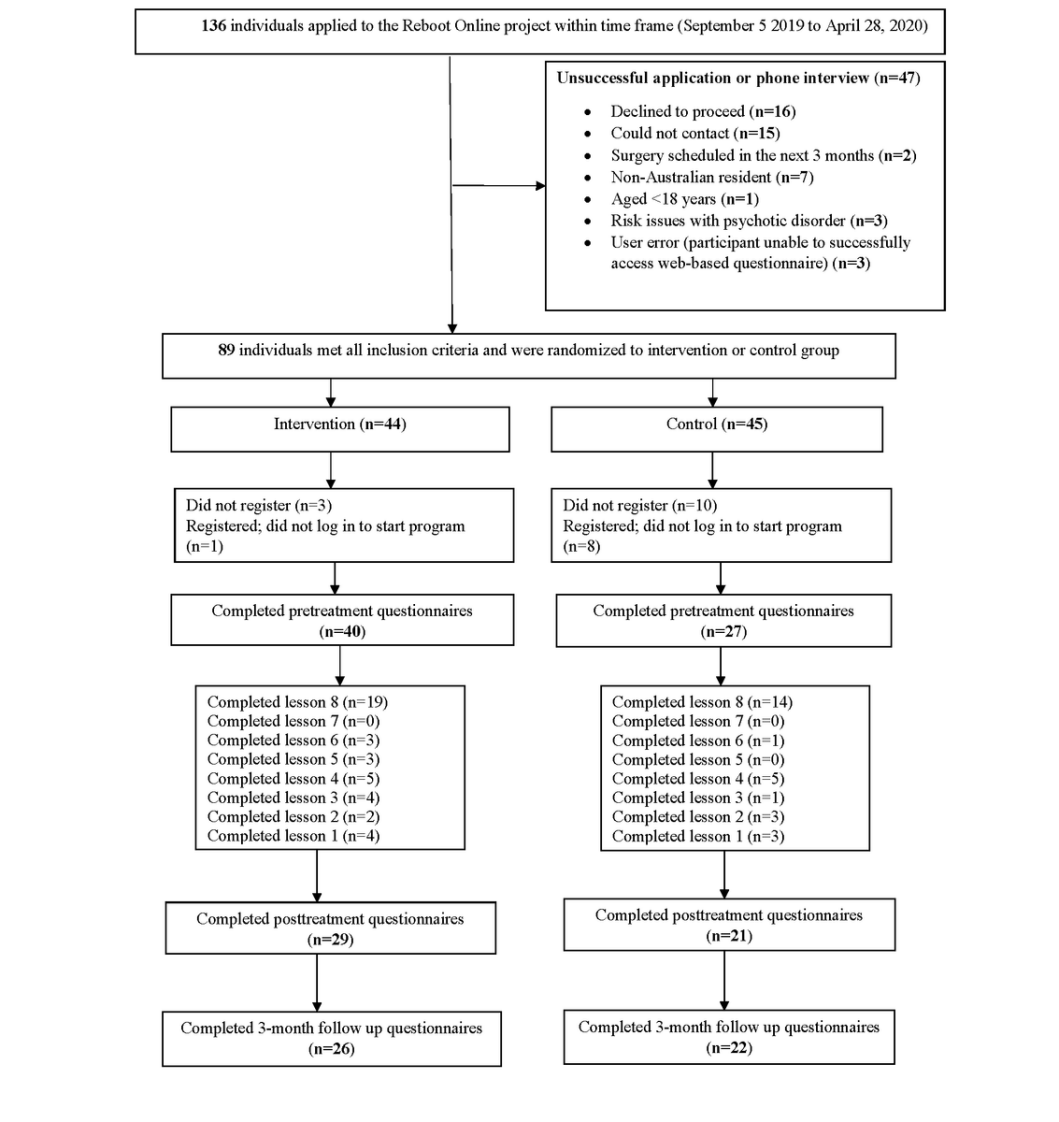
Participant flowchart.

Participant flow through the trial is illustrated in Figure 1. Reboot Online combined with telephone support had a positive effect on enrollment and commencement of the program compared with Reboot Online without telephone support. Significantly more participants from the Reboot Online plus telephone support group enrolled (41/44, 93%) into the course than those from the control group (35/45, 78%; *χ^2^*_1_=4.2; *P*=.04). Furthermore, more participants from the intervention group commenced the course than those from the control group (40/44, 91% vs 27/45, 60%, respectively; *χ^2^*_1_=11.4; *P*=.001).

The median number of lessons completed by those in the intervention versus control groups was significantly different; the intervention group completed a median of 6 (IQR 3-8) lessons compared with a median of 2 (IQR 0-8) lessons in the control group (Mann–Whitney *U*=682; *P*=.009). More participants from the intervention group completed at least half the course (≥4 lessons; *χ^2^*_1_=5.0; *P*=.03) than those from the control group. The overall completion rate for both groups was 37% (33/89). Of the participants enrolled in the intervention group, 43% (19/44) completed the course, and of those in the control group, 31% (14/45) completed the course. When considering the subgroup of those who commenced the program, the overall course completion was 49% (33/67) from both groups; however, there was no significant difference between the proportions of people who completed all 8 lessons in the intervention (19/40, 48%) versus control groups (14/27, 52%; *χ^2^*_1_=1.4; *P*=.24).

### Effectiveness in Clinical Outcomes

Estimated marginal means at pretreatment, posttreatment, and 3-month follow-up as well as within- and between-group effect sizes for all outcome measures, are shown in Table 3. Significant main effects of time showing improvements from before to after treatment were observed for measures of pain self-efficacy (*F*_2,48_=16.4; *P*<.001), kinesiophobia (*F*_2,52_=10.3; *P*<.001), pain interference (*F*_2,53_=12.4; *P*<.001), pain disability (*F*_2,50_=10.8; *P*<.001), and psychological distress (*F*_2,46_=9.6; *P*<.001). There was no significant main effect of time with measures of pain severity (*F*_2,53_=2.0; *P*=.15) or physical activity (*F*_2,55_=1.8; *P*=.19). Within each group, posttreatment improvements were maintained at 3-month follow-up assessment, with no significant changes in outcomes between posttreatment and follow-up values.

There was no significant group-by-time interaction observed for any outcome measure, indicating that treatment efficacy on these outcome measures did not differ between the intervention and control groups.

**Table 3:** Estimated marginal means (EMMs) before and after treatment and at follow-up and within- and between-group effect sizes.

Outcome			Before treatment, EMM (SD)		After treatment, EMM (SD)		Follow-up, EMM (SD)		Pre- to posttreatment within-group comparison					Posttreatment to follow-up within-group comparison					Posttreatment between-group comparison, between-group effect size, Hedges g (95% CI)		Follow-up, between-group comparison, between-group effect size, Hedges g (95% CI)	
									*r*		Within-group effect size, Hedges g (95% CI)		*r*			Within-group effect size, Hedges g (95% CI)						
**PSEQ^a^**																						
	Intervention	26.9 (10.9)		33.9 (12.0)		33.8 (12.3)		0.73		0.61 (0.08 to 1.14)^b^		0.92			0.01 (–0.57 to 0.59)		0.43 (–0.12 to –0.99)					0.32 (–0.26 to –0.89)
	Control	28.7 (10.0)		38.9 (10.5)		37.7 (11.9)		0.37		0.98 (0.28 to 1.67)^b^		0.62			0.10 (–0.55 to 0.76)		N/A^c^					N/A
**TSK^d^**																						
	Intervention	37.4 (6.2)		33.1 (8.0)		33.2 (7.1)		0.60		0.60 (0.07 to 1.12)^b^		0.74			0.01 (–0.57 to 0.58)		0.50 (–0.05 to –1.04)					0.23 (–0.33 to –0.80)
	Control	40.6 (5.9)		36.9 (7.2)		34.8 (7.1)		0.38		0.54 (–0.11 to –1.19)^e^		0.72			0.29 (–0.35 to 0.93)		N/A					N/A
**BPI^f^ severity**																						
	Intervention	5.4 (1.4)		5.1 (1.5)		5.3 (1.4)		0.63		0.25 (–0.27 to –0.76)		0.81			0.19 (–0.39 to 0.77)		0.04 (–0.50 to –0.57)					0.36 (–0.21 to –0.94)
	Control	5.4 (1.3)		5.0 (1.4)		4.8 (1.4)		0.66		0.28 (–0.36 to 0.91)		0.80			0.13 (–0.50 to 0.77)		N/A					N/A
**BPI interference**																						
	Intervention	6.4 (1.8)		5.3 (2.1)		4.9 (2.2)		0.75		0.59 (0.06 to 1.11)^b^		0.78			0.15 (–0.43 to 0.73)		0.01 (–0.5 to –0.55)					0.21 (–0.36 to 0.78)
	Control	6.2 (1.7)		5.2 (1.9)		4.5 (2.2)		0.22		0.53 (–0.12 to –1.18)		0.75			0.36 (–0.28 to 1.00)		N/A					N/A
**PDI^g^**																						
	Intervention	40.1 (12.2)		30.9 (15.6)		31.0 (16.2)		0.69		0.65 (0.12 to 1.18)^b^		0.63			0.01 (–0.57 to 0.59)		0.07 (–0.47 to –0.60)					0.23 (–0.34 to –0.80)
	Control	38.2 (11.5)		31.9 (13.9)		27.3 (15.8)		0.52		0.49 (–0.16 to –1.13)^e^		0.82			0.31 (–0.33 to 0.94)		N/A					N/A
**IPAQ^h^**																						
	Intervention	1571.9 (1709.8)		2783.4 (2790.3)		2353.7 (2391.5)		0.68		0.53 (0.01 to 1.05)^e^		0.56			0.17 (–0.41 to 0.75)		0.18 (–0.37 to –0.74)					0.24 (–0.33 to –0.81)
	Control	2176.6 (1472.6)		2278.5 (2698.4)		1766.6 (2525.9)		0.49		0.05 (–0.54 to –0.64)		0.70			0.19 (–0.40 to 0.78)		N/A					N/A
**K10^i^**																						
	Intervention	27.3 (6.8)		23.2 (7.5)		23.6 (8.7)		0.76		0.57 (0.04 to 1.09)^b^		0.71			0.05 (–0.53 to 0.63)		0.07 (–0.49 to –0.63)					0.02 (–0.54 to –0.59)
	Control	26.4 (5.9)		22.7 (6.3)		23.4 (8.3)		0.40		0.59 (–0.12 to –1.30)^e^		0.71			0.09 (–0.58 to 0.77)		N/A					N/A

^a^PSEQ: Pain Self-Efficacy Questionnaire.

^b^Significance at *P*<.001.

^c^N/A: not applicable.

^d^TSK: Tampa Scale for Kinesiophobia.

^e^Significance at *P*<.05.

^f^BPI: Brief Pain Inventory.

^g^PDI: Pain Disability Index.

^h^IPAQ: International Physical Activity Questionnaire.

^i^K10: Kessler-10 Psychological Distress Scale.

### Telephone Support Sessions

Over the course of the study, all participants in the telephone support group (n=44) had at least one telephone support session and 30% (13/44) had 8 support sessions, with a mean number of 5.7 (SD 2.4) sessions for each participant. The total cumulative telephone time for the intervention group was 3240 minutes, which comprised multiple attempts to make telephone contact as well as successful telephone support sessions, and the overall active telephone time in which the clinician was engaged with the participant was 1780 (54.94%) minutes. This equated to a mean total of 73.6 (SD 31.8) minutes of clinician time per participant over the entire program, of which 40.5 (SD 26.8) minutes was active time spent engaging with the participant. The mean clinician time at each active session was 7.9 (SD 5.5) minutes. No additional time was spent with any participants in either group for follow-up because of acute deterioration in well-being.

### Treatment Satisfaction

When asked to rate their satisfaction with the treatment received in this study on a scale from 0 to 10, the participants provided a median rating of 7 (IQR 5-8). Furthermore, when asked to rate how helpful they found the study intervention for managing their chronic pain, the participants responded with a median rating of 8 (IQR 5-9) out of 10. There was no significant difference between the groups for scores of either satisfaction or how helpful they found the Reboot Online program. Of the 44 participants in the intervention group, 35 (79%) reported that the telephone support made it easier for them to participate in or complete the Reboot Online program. When asked how motivating or encouraging they found the telephone support aspect of their treatment specifically (rated out of 10), the median score of the participants was 7 (IQR 5-9).

## Discussion

### Principal Findings

This study aimed to investigate whether additional clinician guidance, in the form of telephone support, would significantly enhance adherence to the Reboot Online program for chronic pain and improve clinical outcomes. We found that Reboot Online combined with telephone coaching resulted in improved rates of enrollment and commencement (onboarding) of the program compared with the usual Reboot Online program. The overall completion rate from both groups was 37% (33/89; intervention 19/44, 43%, and control 14/45, 31%). The overall completion rate for those who commenced the program from both groups was 49% (33/67); however, there was no significant difference in overall completion rate between the groups (intervention 19/40, 48%; control 14/27, 52%) once the program was commenced. These completion rates are consistent with our real-world adherence outcome of 41% [17] and reflect published attrition rates: 4% to 54% [20]. Furthermore, there was no significant group difference in treatment efficacy on a variety of outcome measures.

The most effective way to provide clinician guidance adjunct to other interventions remains unclear. Little is known as to whether providing guidance evenly timed over the entire course of an intervention is most effective or whether initial support gradually tapered to self-management would be more effective [18]. Our results showed a significant difference between the groups in the rates of enrollment and commencement of the program—the *onboarding phase*. Once participants commenced the program, there was no significant difference in completion rates between the groups, regardless of phone support. This would suggest that telephone support is most effective in improving the onboarding of persons with chronic pain undertaking web-based treatments. The average amount of time involved for each telephone session (including time spent calling to contact participants together with time of consult) was 9.2 (SD 6.0) minutes, with a mean active consult time of 7.9 (SD 5.5) minutes. Thus, the telephone support intervention did not seem onerous and therefore could be easily incorporated into a clinical caseload. When considering where to best use clinical resources, clinician time may be best used in the early phase of onboarding to maximize adherence.

The telephone support provided in this study was delivered by experienced allied health clinicians with specialized training in pain management. The content of the telephone support calls was basic and consisted mainly of reaffirmation of the course material as well as feedback on progress and suggestions to address any barriers that the participants were experiencing. There was no specialized psychological treatment or intervention to address mood or cognition delivered through the calls themselves; nor was there detailed clinical education or advice on movement and physical activity. As the support did not provide specialized intervention, in future it could be provided by a trained peer or junior clinician to streamline the provision of clinical resources, which may be adequate for patients who are not clinically complex. Other studies have shown little difference between clinician and peer-led coaching.

It has been suggested that telephone support provides the possibility to make a diagnosis, tailor the intervention, and actively assist patients to access other needed services [47]. A triage system could also be used to identify persons with more complex issues who need to be referred to specialist clinician intervention. This may be relevant for those with chronic pain who are particularly afraid of movement because of fear of pain because it has been shown that high kinesiophobia negatively effects adherence rates [17]. Research investigating the level of telephone support stratified according to kinesiophobia scores may be worthwhile, with this study being underpowered to investigate this aspect.

The act of individually tailoring a web-based intervention has been suggested to help with adherence and outcomes [47]. Standardized and nontailored internet treatments may leave little room for patient and clinician preferences [48] regarding course content, format, and learning styles. Chien et al [49] identified 5 engagement subtypes in patients enrolled in an internet-delivered CBT program. Their study suggests that by identifying subtypes of patient engagement, programs can be targeted to what is most meaningful to different patients by providing different levels of support or additional treatment modules. The inclusion of telephone support may enable the clinician to assess patient preferences early in the program and adapt the course content to better suit their needs. Others have suggested that allowing the patient to choose the level of support they require would allow efficient allocation of clinician resources to where greater support is wanted, without affecting outcomes or adherence rates [50].

An important consideration related to patient adherence is the logical assumption that adherent patients will have better treatment outcomes than nonadherent patients, although the evidence has not always supported this assumption [51,52]. This study would suggest that additional telephone support does not have an effect on clinical outcomes; however, it did improve onboarding rates, thus increasing the number of participants who commenced the program. Web-based adherence is difficult to monitor because key therapy components are delivered on the web and practiced without the physical presence of a clinician [51]. For instance, a patient may practice the skills outlined in a program without logging in to the internet module. A more relevant way to measure adherence may be to look at intended use, defined as “the extent to which individuals *should* experience the content (of the intervention) to derive maximum benefit from the intervention, as defined or implied by its creators” [52]. This may explain why the patients who did not complete all modules in our study still showed significant improvements in their outcome measures. Further evaluation of the minimum amount of engagement with the program required to induce improvement may provide valuable insight. However, inherent difficulties lie in conducting such an analysis because those who stop engaging with treatments are also more likely to be lost to follow-up assessment.

As internet-delivered interventions evolve, the importance of various technical features in programs and their influence on adherence and outcomes need to be better understood. The mode of guidance, whether by email, text, phone, telehealth, program content, web design, multimedia format, web-based design features, or a hybrid of these features, may play a part in successful engagement of the individual [52]. Qualitative data would support this, with real-world Reboot Online service users anecdotally reporting a desire for more multimedia capability and web-based features. Program developers will need to consider these aspects of design that have an impact on usability and user experience during the development and ongoing evaluation of internet-delivered programs. Further evidence is needed to elucidate what effect each of these design components may have on user engagement and adherence and, in turn, whether design optimization strategies could be successfully used to improve treatment adherence and outcomes [20].

Studies investigating web-based CBT courses for pain conditions [24] report mixed results on whether adjunct guidance improves measures of pain catastrophizing, self-efficacy, and pain interference more than unguided internet interventions alone. Although our study showed significant improvements in measures of pain self-efficacy, kinesiophobia, pain interference, pain disability, and psychological distress over time, there was no significant difference in outcome measures between Reboot Online combined with telephone support and the usual Reboot Online intervention. This may indicate that adding telephone support may not add to the effect of the core Reboot Online program on clinical outcome measures.

The results presented here need to be considered within the context of a number of study limitations. Blinding of participants and the telephone support coach was not possible, introducing a possible risk of bias. Responder bias is another limitation noted because participants self-selected to participate in the trial and outcome data were lost from those participants who dropped out of the course. The power calculation was performed with reference to our primary outcome measure, which was adherence. The study was not powered formally for effectiveness on secondary outcomes and did not correct for multiple comparisons. Loss of follow-up data after treatment and at 3 months resulted in a smaller sample than estimated, which may have led to biased estimates or overestimates of treatment effects. Intention-to-treat analyses were conducted to enable inclusion of participants with suboptimal compliance into the data analysis, and linear mixed model analyses were selected, given that they remain robust in the presence of considerable missing data. Most of the baseline data were collected through patient-reported surveys, and thus the reliability of data are limited by the accuracy of participant recall and self-report. The subjective physical activity questionnaire may have been subject to notable reporting bias; an objective measure of physical activity (such as an activity-monitoring device) is needed in future evaluations’ estimate of physical activity. Although outside the scope of this study, collection of more detailed data on direct intervention costs and opiate use could have facilitated more comprehensive evaluation of the benefits of the Reboot Online program. The study used 2 therapists (TG and JW) to conduct the telephone support, both senior clinicians in chronic pain and trained in the telephone coaching protocol. Although this allowed for consistent intervention during the study, it is unknown whether our findings would generalize to broader clinical settings or multiple clinicians. Finally, the study was conducted between September 2019 and November 2020. During this time period, there were significant and unprecedented local (drought and bushfires in Australia) and global events (COVID-19) that may have had an impact on the ability of participants to adhere and commit to the study as well as on measures of their well-being and mental and physical health.

### Conclusions

Reboot Online offers an effective web-based intervention for chronic pain. Telephone support improves participants’ registration, program commencement, and engagement in the early phase of the internet intervention; however, it did not seem to have an impact on overall course completion or efficacy. To maximize adherence to web-based interventions, clinician resources may be best used in the early phase of onboarding. Further research is warranted to gain better understanding of optimal guidance levels and models as well as program design components that will most effectively improve adherence to web-based interventions.

## Appendix

Multimedia Appendix 1 CONSORT eHEALTH checklist (V 1.6.1).

## Abbreviations

CBT: cognitive behavioral therapy
CONSORT: Consolidated Standards of Reporting Trials
MCID: minimally clinically important difference
MDD: major depressive disorder
REDCap: Research Electronic Data Capture
SMART: specific, measurable, achievable, relevant, and timely
